# A Case Report of Atypical Oral Angiolymphoid Hyperplasia With Eosinophilia (ALHE) Presenting as an Immune Reconstitution Inflammatory Syndrome in a Patient With HIV

**DOI:** 10.1155/crot/6639458

**Published:** 2026-01-10

**Authors:** Rose Haywood, Jeffrey Post

**Affiliations:** ^1^ Infectious Diseases Department, Prince of Wales Hospital, Sydney, Australia, princeofwalesprivatehospital.com.au; ^2^ Microbiology Department, Royal Prince Alfred Hospital, Sydney, Australia, nsw.gov.au; ^3^ University of New South Wales School of Clinical Medicine, Sydney, Australia

**Keywords:** angiolymphoid hyperplasia with eosinophilia, case report, HIV, IRIS

## Abstract

A 35‐year‐old man with uncontrolled human immunodeficiency virus (HIV) infection presented with a pharyngeal plaque‐like lesion after recommencing antiretroviral treatment (ART). Rapid localized growth of the lesion paralleled his rising CD4+ T‐cell count, suggesting immune reconstitution–mediated deterioration. The diagnosis of the lesion as angiolymphoid hyperplasia with eosinophilia was made more challenging by the atypical immune response that accompanies the HIV‐associated immune reconstitution inflammatory syndrome.

## 1. Introduction

Angiolymphoid hyperplasia with eosinophilia (ALHE) is a rare condition of inflammatory tumefaction characterized by vascular proliferation and dense lymphocytic and eosinophilic infiltrate. ALHE can present on mucosal surfaces but most commonly presents as cutaneous papules or nodules on the head, particularly in the peri‐auricular region. Its pathogenesis remains unknown as its varied presentations may reflect either reactive or neoplastic processes. During immune reconstitution, a period characterized by abnormal inflammatory responses and atypical manifestations of immune reactivity, the diagnosis of ALHE requires an index of suspicion.

## 2. Case Presentation

A 35‐year‐old man with human immunodeficiency virus (HIV) and hepatitis C virus coinfection presented with an atypical pharyngeal lesion. He had a history of intravenous drug use and cigarette smoking. He had recently recorded a CD4+ T‐cell count nadir below 100 cells/*μ*L due to self‐cessation of antiretroviral therapy (ART). Three months prior to the lesion appearing, he had been recommenced ART, which included emtricitabine, tenofovir disoproxil fumarate, and ritonavir‐boosted darunavir.

A small plaque‐like lesion was first noted near his right lower wisdom tooth. As the lesion resembled oral hairy leukoplakia, he was treated with courses of valaciclovir and aciclovir while the lesion relapsed and remitted. After four months, his CD4+ T‐cell count had risen to 340 cells/*μ*L and HIV replication was fully suppressed, but this was accompanied by growth of the thick, white, well‐circumscribed, exophytic plaque to cover the left tonsil, along with uvula enlargement, a change in voice quality, a dry cough, and bilateral otalgia. There was no lymphadenopathy nor systemic symptoms.

**Figure 1:**
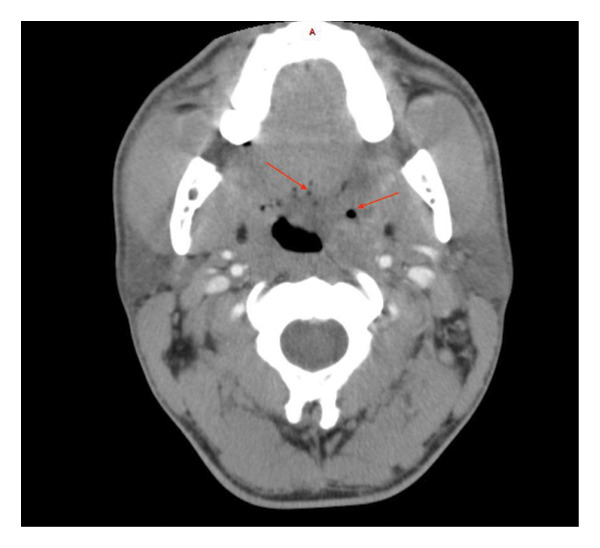
CT head with intravenous contrast demonstrating oropharyngeal lesion with areas of necrosis (red arrows).

The patient had mild eosinophilia (0.46 × 10^9^/L) with normal levels of other leucocytes. Syphilis serology was negative. A CT scan (Figure 1) demonstrated that the lesion extended from the nasopharynx to the oropharynx. He was commenced on amoxicillin/clavulanic acid orally with no change in the lesion. A left tonsillar biopsy was performed which was complicated by postoperative hemorrhage.

**Figure 2:**
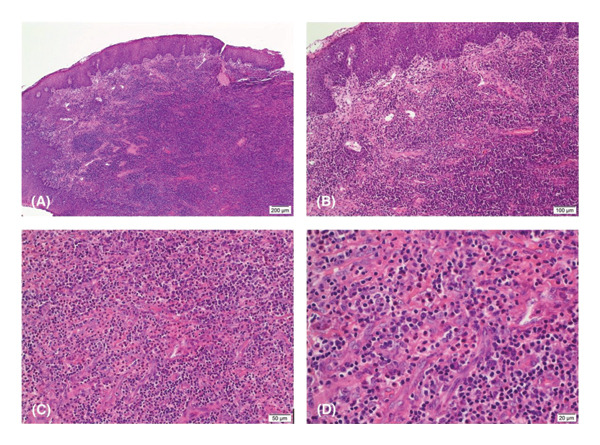
(A–D) Histology demonstrating (A) dense inflammatory infiltrate within submucosal stroma, (B) with extension into overlying squamous epithelium, and (C) eosinophil‐rich inflammation, (D) with proliferation of small‐caliber vessels lined by plump endothelium.

Microscopic examination (Figures 2(A), 2(B), 2(C), and 2(D)) of the tonsil revealed a dense inflammatory cell infiltrate consisting largely of plasmacytoid lymphocytes, plasma cells, and eosinophils. There was exocytosis of eosinophils into the overlying squamous mucosa and vascular proliferation comprising of numerous thin‐walled capillary‐sized vessels arranged in a lobular pattern. Foci of necrosis were present, some of which were centered around blood vessels, but there was no vasculitis. The B‐ and T‐cell markers were of a benign mixed pattern, excluding a lymphoproliferative process.

The biopsy demonstrated a reactive process with unusual areas of necrosis, although no opportunistic infection was identified: no fungi were seen on the methenamine silver or periodic acid–Schiff stains; the Warthin–Starry stain for spirochetes was negative; no acid‐fast bacilli were seen; and *Cytomegalovirus*, herpes simplex virus, and LMP1 for Epstein–Barr virus immunoperoxidase stains were negative. The vascular proliferation and eosinophilia were most consistent with angiolymphoid hyperplasia with eosinophilia; however, the vessels lacked the classic cobblestone endothelial lining, and the location in the tonsil was not typical. The presence of eosinophilia, along with the clinical picture, distinguishes this from pyogenic granuloma, which is seen on the oral mucosa but is characterized by a classic red polypoid appearance.

Despite various treatments including antibiotics, antivirals, and surgical debridement, the lesion continued to wax and wane, but after 48 months, it resolved completely, possibly due to immune control.

## 3. Discussion

There have been a number of reports of ALHE presenting in patients with HIV, but it has not previously been described in the context of immune reconstitution inflammatory syndrome (IRIS). IRIS is the paradoxical worsening or new clinical manifestation of an underlying infection or autoimmune disease that accompanies a restoration of the immune system. The commencement of effective ART precipitates a rapid recovery of CD4+ T cells, so IRIS commonly manifests within the first 3 months of commencing ART. The diagnosis of IRIS requires an initial presentation or exacerbation of an infective, neoplastic, or inflammatory process following the commencement of effective ART and the associated decrease in plasma HIV RNA by greater than 1 log_10_ copies/mL or increase in CD4+ T‐cell count. The presentation should be atypical or exaggerated, and spontaneous resolution may occur with continuation of ART [1]. The case presented here met this case definition.

Whilst IRIS is more commonly associated with infections such as mycobacterial or fungal infection, it has also been encountered in patients with autoimmune disease including thyroid disorders and vasculitis. Neoplastic processes accelerated by IRIS are epitomized by the presentation of Kaposi sarcoma soon after the commencement of ART.

The pathogenesis of ALHE remains unknown, but favored hypotheses include a reactive process in the context of trauma [2], an infective process such as an association directly with HIV [3], or a neoplastic process with benign vasoproliferation [4].

ALHE closely resembles Kimura disease, though the latter is typically characterized by lymphadenopathy, peripheral eosinophilia, and elevated IgE, which were not seen in this case [5]. Diagnosis of ALHE is made via biopsy of a suspicious lesion which classically presents as a papule or nodule on the head or neck, rarely on mucosal surfaces. Histology typically demonstrates proliferative tufts of capillaries lined by plump endothelial cells which project into the vascular lumen (“cobblestone appearance”), hence why it is also known as epithelioid hemoangioma [3]. The surrounding stroma shows an absence of fibrosis. The condition is distinguished histopathologically from Kimura disease, which is characterized by the presence of lymphoid follicles [4].

In keeping with the enigmatic nature of this rare condition, recommended treatment modalities vary, including photodynamic therapy, intralesional chemotherapy, and surgical excision, amongst others. Whilst it is a benign condition, local recurrence is common regardless of the therapeutic approach. Such marked deterioration as seen in the above case report has not been previously reported.

## 4. Conclusion

An ALHE lesion associated with CD4 T‐cell aberrancies could very well manifest a primary presentation during the period of immune reconstitution and restoration of CD4 T cells, as with an IRIS reaction. The atypical picture in this case, notably the unusual site and the irregular areas of necrosis, is in keeping with an aberrant immune response. The unusual manifestation of a new condition and the temporal association with the rapid complete suppression of HIV replication and associated restoration of CD4+ T cells are consistent with an IRIS response.

## Consent

Informed consent to publish pertinent case information and images was obtained from the patient.

## Conflicts of Interest

The authors declare no conflicts of interest.

## Author Contributions

Rose Haywood completed the primary manuscript writing and necessary edits. Jeffrey Post provided critical review and supervision.

## Funding

No funding was received for this case report.
